# Co-creating with patients an impact framework across the medicine’s life cycle: a qualitative study exploring patients’ experiences of involvement in and perceptions of impact measures

**DOI:** 10.1186/s40900-022-00334-0

**Published:** 2022-02-02

**Authors:** Oleks Gorbenko, Pascale Cavillon, Rachel H. Giles, Teodora Kolarova, Muriël Marks, Antonella Cardone, Sandeep Bagga, Claire Nolan

**Affiliations:** 1 Ipsen, Slough, Berkshire, UK; 2 Ipsen, Boulogne-Billancourt, France; 3 International Kidney Cancer Coalition (IKCC), Duivendrecht, The Netherlands; 4 International Neuroendocrine Cancer Alliance (INCA), Boston, USA; 5 World Alliance of Pituitary Organisations (WAPO), Amsterdam, The Netherlands; 6 European Cancer Patient Coalition (ECPC), Brussels, Belgium; 7 MediPaCe, Maidenhead, UK

**Keywords:** Patient engagement, Patient participation, Patient centricity, Impact, Return on engagement, Medicines development

## Abstract

**Background:**

The biopharmaceutical industry is challenged with efficiently delivering medicines that patients truly value. This can be addressed by engaging patients and caregivers throughout a medicine’s life cycle, ensuring that products meet the needs and expectations of those who take them. While isolated best practice examples of patient engagement exist, they remain relatively ad hoc and not fully embedded within Research & Development (R&D) practices. To encourage more patient engagement, the ‘impact’ of patient engagement projects (PEP) must be objectively measured and demonstrated. Some frameworks have been proposed; however, there is no evidence of widespread adoption, nor have patients’ perspectives been robustly explored. The objective of this qualitative study was therefore to understand patients’ perspectives of impact measurement that can be systematically applied within a biopharmaceutical company.

**Methods:**

Semi-structured interviews were conducted with 13 patient organisation (PO) representatives exploring their experiences of engagement and reflections on 23 candidate patient engagement impact measures categorised into five groups: Medicines R&D Priorities; Clinical Trial Design; Regulatory & Market Access Submissions; Product Support & Information; and Disease Support & Information. Thematic analysis was undertaken and impact measures revised in line with interview participant feedback. Emerging themes and revisions to impact measures were validated at a joint workshop with 4 patient advisors representing 4 POs.

**Results:**

The study revealed that PO representatives feel a deep sense of accomplishment and ownership when collaborating on PEPs with biopharmaceutical companies. They largely conceptualise ‘impact’ as positive, tangible and useful outcomes. The revisions made to the pre-defined patient engagement impact measures fell into three broad categories: (1) a requirement for greater context; (2) capturing the nature of patient influence; and (3) terminology changes. The greatest number of revisions concerned ‘requiring greater context’, for example, including additional descriptions, patient quotes, and satisfaction.

**Conclusions:**

This study sheds light on how patient advocates view ‘impact’. Typically this means delivering ‘value’ important for them. Therefore, the authors of this paper created the term ‘value-impact’ to comprehensively characterise this conceptualisation, and propose a value-impact measurement plan, incorporating longitudinal data. Through this understanding and in light of other recently published work, wide-scale adoption and implementation of the measurement of value-impact across the biopharmaceutical industry can be realised.

## Plain English summary

Developing medicines involves multiple stages, all essential for pharmaceutical companies. However, this approach is becoming increasingly costly and patients are rarely involved in defining treatment outcomes. Patient insight is important for everyone involved in medicinal development and required by authorities reviewing new treatments. To identify best practices, the difference (‘impact’) made by engaging with patients must be measured continuously throughout the treatment development process. Possible impact measures have been proposed (for example, the PARADIGM initiative), but problems remain.

This study aimed to work with patients and adapt standard measuring approaches that can be used to provide ongoing insight into the impact of patient engagement activities for companies and patients. Researchers interviewed 13 patient organisation (PO) representatives to explore their experiences of impact measures. Thematic analysis was conducted, and data used to adapt 23 potential impact measures in collaboration with patients.

This study identified that PO representatives view impact as being positive, tangible, and useful outcomes, achieving a deep sense of accomplishment and ownership for those patients and representatives involved. The authors created the term ‘value-impact’ to describe how patients perceive such achievements. Furthermore, measures should be adapted to: 1) include greater context such as descriptions, patients quotes and satisfaction; 2) capture patient influence; and 3) use language that is understandable for all parties. Examples and suggestions for engagement at different stages and revised impact measures are presented in this report. It is our hope that this study will encourage more frequent and effective use of patient engagement strategies as medicines are developed.

## Introduction

Over recent decades, global healthcare has begun to demonstrate a greater focus on the patient as the final user of health technologies and beneficiary of the value associated with their adoption. Several healthcare stakeholders increasingly consider unmet patient needs to be a crucial driver across the health technologies’ life-cycle and national healthcare systems [1]. As one of these stakeholders, for the biopharmaceutical industry, adopting such a patient centric approach is not an entirely new concept [2]. 

However, the biopharmaceutical industry has not yet fully embedded this concept extensively within all processes and operations supporting the medicines life-cycle or as a critical component of corporate culture. The picture today still consists of a largely product-focused business model that leverages product-centric attributes, rather than value-centric attributes important to the patient. In this product-focused model, resource emphasis has been on addressing the needs of those stakeholders upstream of the patient end-user; the regulator, the payor and the healthcare provider. Furthermore, the cost in bringing potential new medicines through discovery, pre-clinical, and clinical development and regulatory approval continues to appreciate. In the 1980s the cost to bring a medicine to market was estimated at $231 M [3]. More recently the cost of developing a prescription drug that gains market approval has been estimated at $2800 M [4]. In a post Covid-19 world with an impending global recession, society’s ability to sustain these costs will be increasingly scrutinised [5]. The industry can ill afford to advance potential new medicines if they do not demonstrate real value to the patient and to society. Therefore, the challenge for the biopharmaceutical industry is to deliver medicines that patients truly value, to do this more efficiently and to make the entire medicines life cycle more patient-centric.

These risks of delayed R&D and under-valued medicines could be successfully mitigated by holistic, timely, compliant engagement and collaboration with patients, caregivers, advocates, and community representatives throughout a medicine’s life cycle ensuring continuous input on unmet needs and expectations. We have seen a shift in emphasis, by the regulator in particular, from a product-focused approach to a more patient-focused and value-driven drug development process, with the Food and Drug Administration (FDA), European Medicines Agency (EMA) and Medicines and Healthcare products Regulatory Agency (MHRA) making progress in incorporating the patient perspective into medicine review requirements [6–12]. Some national healthcare systems have also transformed Health Technology Assessments (HTA) from traditional incremental cost-effectiveness evaluation models towards complex value-based assessments (VBA), with more requirements to demonstrate value using Patient Reported Outcomes Measurements (PROM) and improvements within health-related quality-of-life (HRQoL) [6, 13, 14]). Initiation of such input as early as possible in a medicine’s life-cycle has become, therefore, a critical success factor for the industry [15–17]).

There are some best practice examples and case studies of effective patient engagement occurring across a medicine’s life-cycle that have delivered a real value and impact on the patient’s condition [18–20]. These examples are limited in number and unequally distributed across a medicine’s life-cycle – from least explored: discovery and early development, to the most explored: clinical operations and post-authorisation [21]. Against this backdrop, some research has shown there is a prevalent view that patient expertise is little appreciated [22]. Patient engagement activity still appears to remain relatively ad hoc and not fully embedded within R&D practices. Therefore, in order to shift the status quo and encourage more patient engagement, the ‘impact’ achieved from patient engagement needs to be objectively measured and demonstrated.

A recent literature review funded by the PARADIGM group highlighted the opportunity to monitor and evaluate patient engagement with a proposed list of impact measures and methods [23]. The authors have recently released a comprehensive and extensive framework of impact measures [24]. Other metrics guidance overviews have been generated along with past and existing frameworks and conceptual models that make reference to ‘impact’, ‘assessing participant engagement’ or ‘performance’ however without evidence of adoption yet [16, 25–38]. Furthermore, in recent years, theoretical financial benefits of earlier patient involvement delivering more effective and efficient cost-saving R&D have been posited [39, 40]. The key elements and recommendations on how impact should be evaluated reflect the extensive debates, experience and work done by the authors and their contribution to the development of these conceptual models and frameworks.

Remarkably, the patients’ perspective on patient-centric impact measures is largely missing in the literature. For instance, there are no studies, to the authors’ knowledge, that systematically capture patients’ opinions and perceptions on a range of patient engagement impact measures or explore their experiences of impact measurement following involvement activity.

There is a common need for the biopharmaceutical industry to elaborate a clear, unified approach for measuring the impact of patient engagement activities. Continuous patient input and co-working are critical for this purpose as well as cross-functional involvement at the corporate level. Referring to best practice examples and cases from across the biopharmaceutical industry alone cannot be considered as an optimal solution and should only be taken to support a proposed approach and methodology.

After recognising a gap within their own internal processes, and acknowledging a common need across the industry, Ipsen has launched this research initiative to contribute to the industry-wide/cross-sectoral debate on meaningful impact measurement of patient engagement projects (PEPs). Therefore, the objective of this qualitative study is to understand patients’ preferences and expectations of impact measurement in the context of their experiences of patient engagement activity. In particular, this paper describes patients’ perspectives on what are meaningful measures of impact that could help create the momentum to turn ad hoc patient engagement practice into routine, standard practice within the biopharmaceutical industry.

## Methods

This study adopted a qualitative research design to explore the views and experiences of the patient community with impact and impact measurement while working with biopharmaceutical companies. The primary research instrument used was semi-structured interviews which allow for an extended and deeper exploration of people’s opinions while permitting opportunities to probe unanticipated topics, use subtle prompts and checks for accuracy and relevance. In addition, with the interviewees based across the world, interviews are also logistically easier to arrange. The consolidated criteria for reporting qualitative studies (COREQ) have been used to report the study [52].

**Fig. 1:**
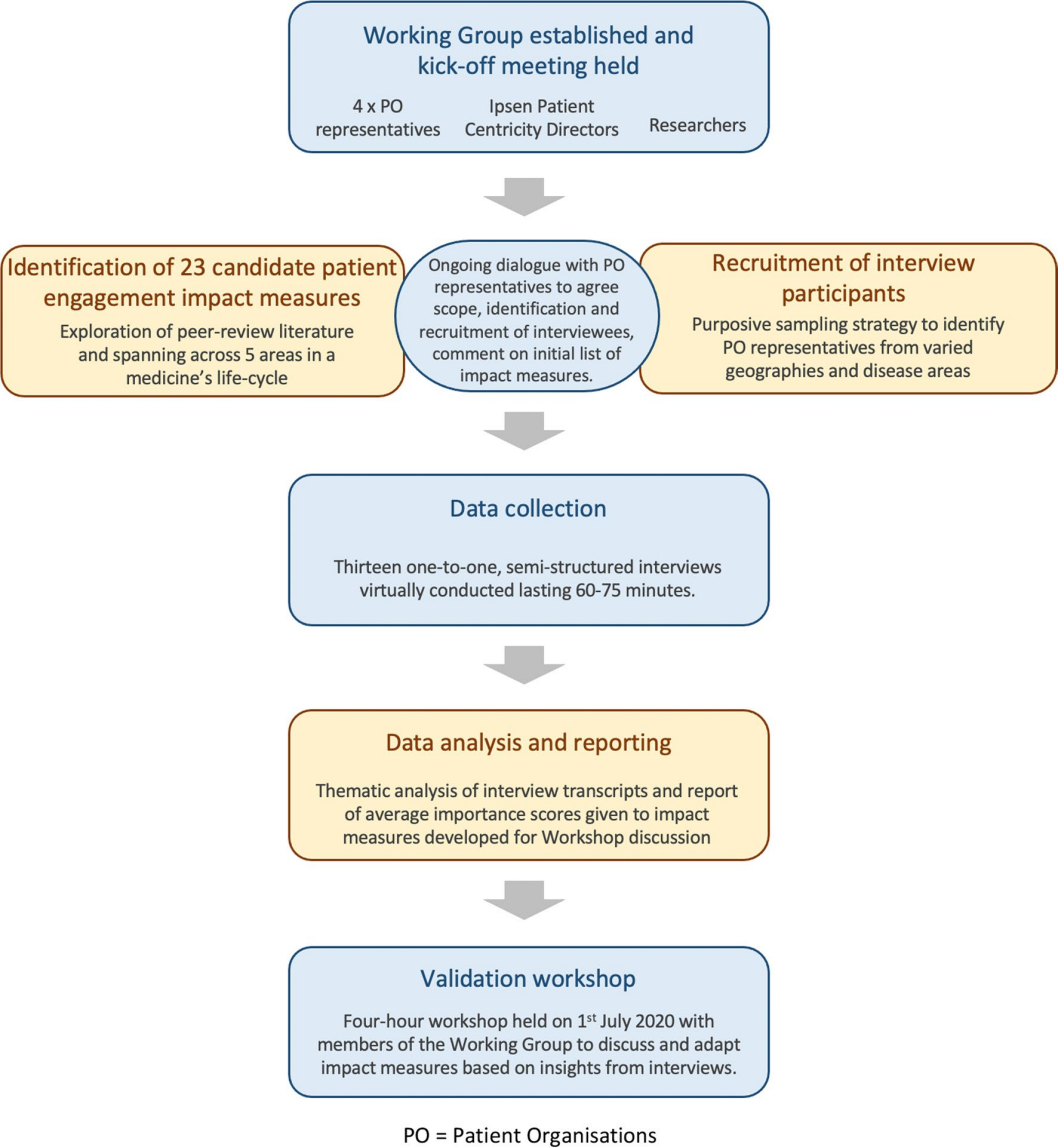
Overview of research process. *PO* patient organisations

The study consisted of three stages: (i) semi-structured interviews with Patient Organisation (PO) representatives exploring their experiences of involvement in, and reflections on, a series of impact measures; (ii) thematic analysis and revision of impact measures in line with interview participant feedback; (iii) validation workshop to discuss the data and make recommendations for final revision and practical implementation of impact measures. Figure 1 provides an overview of the research process undertaken.

This section describes the formation and role of the study’s Working Group, and delivery of the semi-structured interviews and workshop with PO advisors. Table 1 lists definitions of terms commonly used by the authors during the study.

**Table 1:** Table of definitions

Term or concept	Definition
Impact (from patient-centric projects)	Broader effect of outcomes, both positive and negative of patient engagement which could be either indirect or direct, intended or unintended [23]
Life science industry	Standard Industry Classification (SIC) codes (specifically SIC 2007) define life science industry as an industry represented by companies whose primary activity is manufacturing (e.g. pharmaceuticals and medical devices) or those whose primary activity is research in biotechnology (which includes non-health biotechnology). The broader definition comprises companies operating in the research, development and manufacturing of pharmaceuticals, biotechnology-based food and medicines, medical devices, biomedical technologies, nutraceuticals, cosmeceuticals, food processing, and other products that improve the lives of organisms [50, 51]
Medicine’s life cycle	The process of drug development from discovery, through early research into development and ultimate utilisation of the medicine in clinical practice [41]
Patient	A person who lives with a health issue, risk and/or disease. In our paper, this definition includes the person who has the medical condition, as well as those who live with and/or care for this person (i.e. family and caregivers)
Patient engagement project (PEP)	Subject matter collaboration between patients, patient advocates, patient representatives, caregivers and the industry in the processes and decisions within the medicines life cycle (see Patient Engagement). According to the Patient Engagement Value Model, PEP should include listening, insights generation and translation, co-creation and measuring impact
Patient Engagement	The effective and active collaboration of patients, patient advocates, patient representatives and/or caregivers in the processes and decisions within the medicines life cycle, along with all other relevant stakeholders when appropriate [42]
Patient Expert	A relatively new category of external non-HCP experts who meet the key criteria of appropriate capabilities (subject matter expertise) and representation, or ability to represent views and interests of many patients and/or patient organisations. Patient expertise may also manifest as the achievement and/or demonstration of academic/scientific influence, such as an EUPATI fellowship [43, 44]
Patient Involvement	Used synonymously/interchangeably with ‘Patient Engagement’, though ‘involvement’ suggests a more active and collaborative engagement to understand the patient perspective [53]
Patient Organisation (PO)	A collaborative group of individuals that provides patient support and/or lobbies on behalf of the collective views of patients, where patients and/or caregivers represent the majority of members in a governing body [43]
Return on Patient Engagement	The impact derived as a result of a patient engagement activity [23]
Value (from patient engagement projects)	The benefits of patient engagement for individuals or organisations involved [23]

### Working group

Members of Ipsen’s Patient Advisory Board were invited to take part as advisors and co-authors for the present study. The Patient Advisory Board consists of senior leaders of national and international POs. Four PO representatives agreed to participate in the Working Group. Other members of the Working Group included Ipsen’s Patient Centricity Directors and the research investigators (Table 2). The composition of the Working Group ensures equal voice and representation from the patient community and other stakeholders throughout all stages of the study.

**Table 2:** Members of collaborative Working Group

Organisation	Position
International Kidney Cancer Coalition (IKCC)	Founder, Chairman of the Board
European Cancer Patient Coalition (ECPC)	Director
World Alliance of Pituitary Organisations (WAPO)	Executive Director
International Neuroendocrine Cancer Alliance (INCA)	Executive Director
IPSEN	Global Patient Centricity Director
IPSEN	Global Patient Centricity Director
MediPaCe	Patient Expert
MediPaCe	Patient Advocacy Lead
MediPaCe	Research Lead

The Working Group’s primary role included:

- Monitor study progress and help resolve any challenges or blockers
- Review and approve core research material (e.g. interview discussion guide)
- Review and analyse findings from semi-structured interviews
- Attend validation workshop and take part in the deep-dive discussions
- Follow-up review and finalisation and impact measures

An initial meeting was held on 27^th^ April 2020 to discuss and finalise the study’s remit in addition to the proposed methodology.

### Identification of impact measures

A list of 23 candidate impact measures, categorised into 5 groups, that could be used to evaluate patient engagement was collated for Ipsen in a previous unpublished undertaking by the study researchers based on a scoping review of peer-reviewed literature and deep-dive analysis of the selected 15 patient engagement frameworks and conceptual models. As described above, many of these frameworks and models have a well-represented patient vision and expectations on how impact should be defined and measured and also reflect the authors’ experience with them [18, 25–27, 29]. The initial list of candidate impact measures was refined during ongoing dialogue with members of the Working Group. For the present study, this list was considered to be a reasonable foundation upon which to obtain participant views and opinions. In keeping with PARADIGM’s recognition that the impact of patient engagement is likely to differ at different decision-making points in the medicines life cycle, we categorised our original impact measures accordingly [23]: *Medicines R&D Priorities*; *Clinical Trial Design*; and *Regulatory/Market Access Submission*. In addition, we also wanted interview participants to reflect on patient engagement during the co-creation of patient support programmes, therefore two additional categories were included: *Product Support and Information*; and *Disease Support and Information* (Table 3).

**Table 3:** Original list of 23 patient engagement impact measures

Medicines R&D priorities	Clinical trial design	Regulatory/market access submission	Product support and information	Disease support and information
Number of changes made to the research or development plan	Number of changes to reduce the burden of study for patient participants	Patient insights included in development programme to inform submissions	Patient understanding of their medicine	Improved engagement with their disease and/or ability to self-manage
Development of clinical outcomes/clinical measures	Earlier regulatory submission, approval and/or market access submission	Patients’ critique of evidence generated from clinical trials included in submission	Patient adherence with medicine	Patient adherence with medicine
Development of Patient Reported Outcomes/Experiences	Number of changes made to the final version of patient-facing documents	Achieving regulatory approval/market access recommendation consistent with patient population studied	Clinical outcome or clinical measure improvements	Clinical outcome or clinical measure improvements
Development of tolerability/side effect profile	Number of patients complying with study protocol	Achieving regulatory approval/market access recommendation with more informed label	Patient opinions on risk/benefit	Reduction in utilisation of healthcare resources
Patient testimonials/feedback*	Study participants’ experience & satisfaction ratings		Reduction in utilisation of healthcare resources	

*Patient testimonials/*feedback* is displayed only once however applies to all five stages

### Semi-structured interviews

Participants were recruited with the support of Ipsen’s Patient Advisory Board members and through the researchers’ existing contacts with a PO network, via email correspondence. A purposive sampling strategy was used to approach individuals who met the agreed participant criteria outlined in Table 4. This criteria was developed in agreement with the Working Group to ensure those interviewed had the necessary experience and/or knowledge to be able to provide views on impact measurement. ‘Leadership’ within the patient community or ‘representation’ were considered the 2 most fundamental criteria. Of the 16 people contacted, 13 were available for interview. Two declined because they disagreed with the reimbursement for participation. One other declined as they did not have time to take part. In agreement with the Working Group, efforts were made to ensure diversity of opinions and experiences. Therefore, the researchers coordinated efforts to approach individuals from a wide range of geographical locations (across the world), type of PO (country, regional, umbrella/global), and wide range of disease areas (Table 5). On the latter point, it was agreed disease areas should also include those that Ipsen is not currently operating in or exploring to achieve a wider representation. With the aim to avoid bias, the 4 PO members of the Working Group did not take part in the interviews. Table 4 outlines the agreed participant criteria for inclusion.

**Table 4:** Core criteria for interview participants

***EITHER* Leadership *OR* Representation** ***AND* two of Capabilities, Impact or Collaboration** Leadership: Seniority and recognised leadership position within their country, regional or global PO (Head, Director, Vice President, President or the equivalent, board member) and broader patient community Representation: Connected to and representative of a significant number of patients and/or POs; ability to speak on behalf of them representing their interests Capabilities: Knowledge, literacy, advocacy training and/or experience of medicines development process (e.g. knowledge and/or experience of clinical trial protocol development or patient support programme development, review of any R&D documents and contribution to R&D/Medical advisory boards, membership in Community Advisory Boards) and/or EUPATI, EORTC or other R&D training programme Impact: Knowledge, activism and expertise on impact and value of patient engagement Collaboration: Experience of working with pharmaceutical companies, regulatory agencies and/or HTA bodies as well as contribution to cross-sectoral initiatives (e.g. PFMD, PARADIGM, INVOLVE, NHC etc.)	

PO = Patient Organisation, EUPATI = European Patients’ Academy, EORTC = European Organisation for Research and Treatment of Cancer, PFMD = Patient Focused Medicine Development, INVOLVE = a public participation charity, US NHC = National Health Council

**Table 5:** Sample characteristics of qualitative interview participants

Characteristic	(*n* = 13)
PO representative	12
Patient Expert *(operates independently)*	1
Type of PO	
Umbrella/global	2
Regional	4
Country	5
Geography	
UK	1
US	4
Germany	1
Argentina	1
Australia	1
Malaysia	1
Belgium	2
France	1
Italy	1
Therapeutic areas	
Neuroendocrine	1
Acromegaly	1
Prostate cancer	1
Bladder cancer	2
Parkinson’s disease	1
Neurology *(umbrella area)*	1
Multiple sclerosis	1
Duchenne Muscular Dystrophy	1
Alkaptonuria	1

*PO* Patient organisation

An interview discussion guide was developed by the research investigators and refined by the working group. The first part of the interview consisted of a set of root questions covering participants’ experiences and perspectives on patient engagement impact measurement, each followed by a set of prompts and probes to explore additional detail. The second part focused on the impact measures listed in Table 2. In order to realistically and practically obtain useful feedback on each individual measure, an accompanying PowerPoint slide deck was developed displaying each measure with a rating scale. During the interviews, participants were asked to indicate how important they thought each impact measure was (0 = not important; 5 = very important) and to provide their reasoning. Discussions explored participants’ general experiences within the Life Sciences Industry without focusing on any one particular company.

Thirteen participants took part in one-to-one, semi-structured interviews conducted between 3rd and 19th June 2020. Online interviews were conducted by two researchers, SB (male, trained moderator) and CN (female, trained moderator), using the Zoom video-conferencing platform. Both interviewers have significant experience generating qualitative insights on patients’ lived experiences. The interviews were audio-recorded and lasted 60–75 min. Participant information sheets were sent to interviewees and informed consent was recorded prior to the start of the interviews.

Key ethical considerations included ensuring that individuals were aware that their participation was entirely voluntary – not coerced in anyway and that they had all the information necessary to fully consider taking part (e.g. purpose, sponsor, time required, complaints procedure with contact details provided). This was clarified in the participant information sheet and then again before starting the interview itself, with another opportunity to ask questions. The other consideration was about the data provided by participants. With regards personal data, the rights of participants were clarified prior to the interview. It was made clear that the audio-recordings would be deleted as soon as verbatim transcripts were prepared (done immediately) and that the transcripts would be pseudonymised. In addition, any comments made would not be attributed to the participant or the PO that they represent i.e. to remain completely anonymous. It was also made explicitly clear upon the start of the interview that interviewees need not mention any biopharmaceutical company that they had worked with nor any commercially sensitive information.

### Analysis

All interview recordings were transcribed verbatim and assigned a unique serial number. Audio-recordings were subsequently deleted. Thematic analysis based on Braun and Clarke’s [45] framework was used to analyse the qualitative data and to identify recurring themes related to patient engagement experience [45]. The process of data familiarisation took place during data collection and transcribing. Initial transcripts were individually coded (identifying units of meaning) by two researchers who then reviewed the other’s codes. Through subsequent discussion and reflection, codes were finalised and applied throughout the remaining transcripts. Through an iterative process, codes were further categorised to form themes.

### Validation workshop

A comprehensive report consisting of identified themes and interview participants’ feedback on impact measures was shared as a pre-read with members of the Working Group. PO advisors in the Working Group provided comments prior to the workshop. This pre-workshop feedback was particularly valuable in helping to focus discussions and drive recommendations during the workshop.

The 4-h virtual workshop was held online on 1^st^ July 2020 during which PO advisors had significant involvement and rigorous discussions aimed at adapting and refining impact measures to ensure they are meaningful from the patient’s perspective and could be feasibly implemented. Each individual impact measure was displayed along with the following information: average importance score (0–5), range of scores, ranking against others within the category (stage of medicines’ life cycle), interviewee quotes that supported the measure and those that were critical of a particular aspect and comments received from the 4 Working Group patient advisors (pre-workshop feedback). One researcher moderated discussion and the other provided additional details and nuance as necessary and kept a record of discussions and final decisions.

**Fig. 2:**
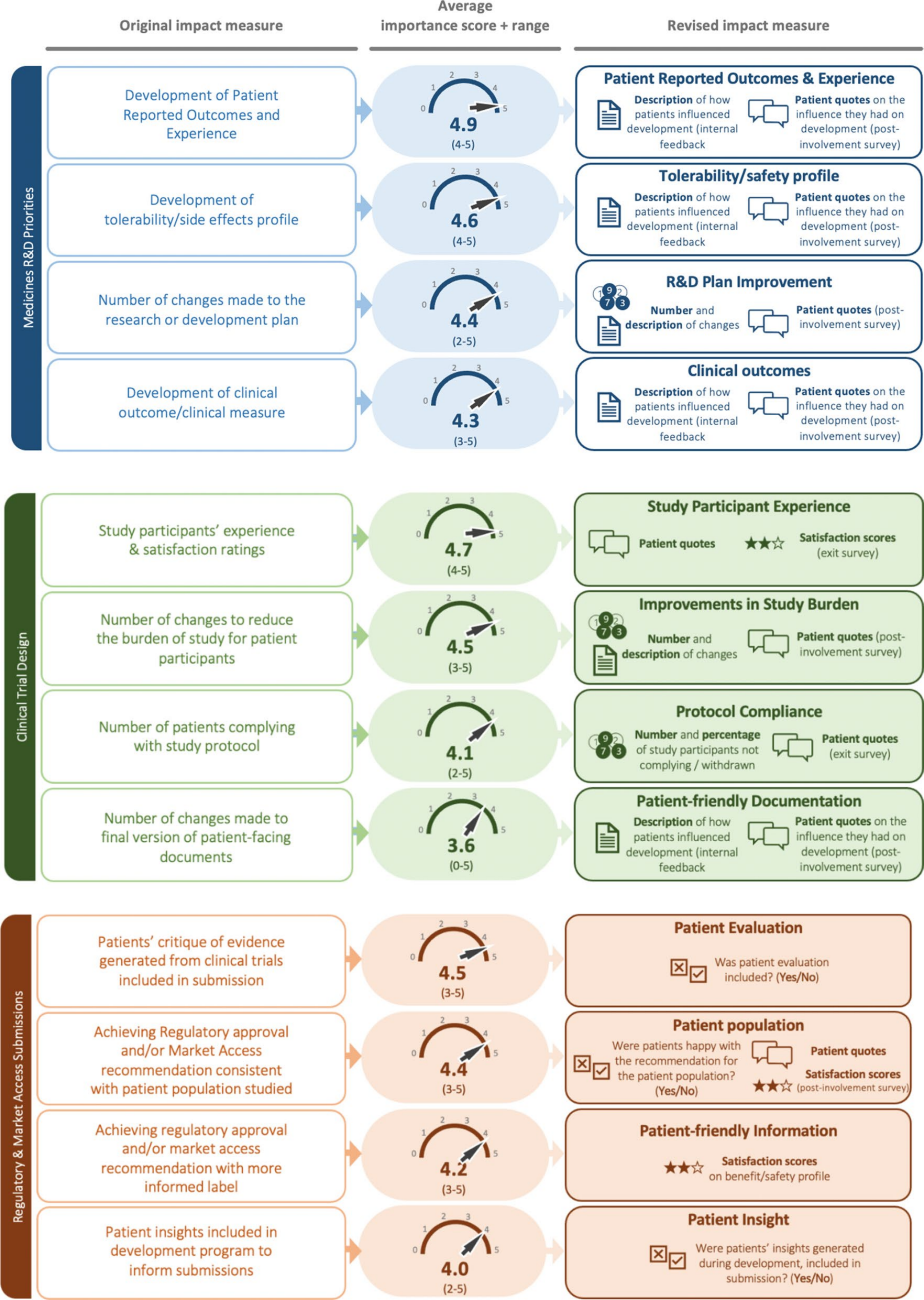

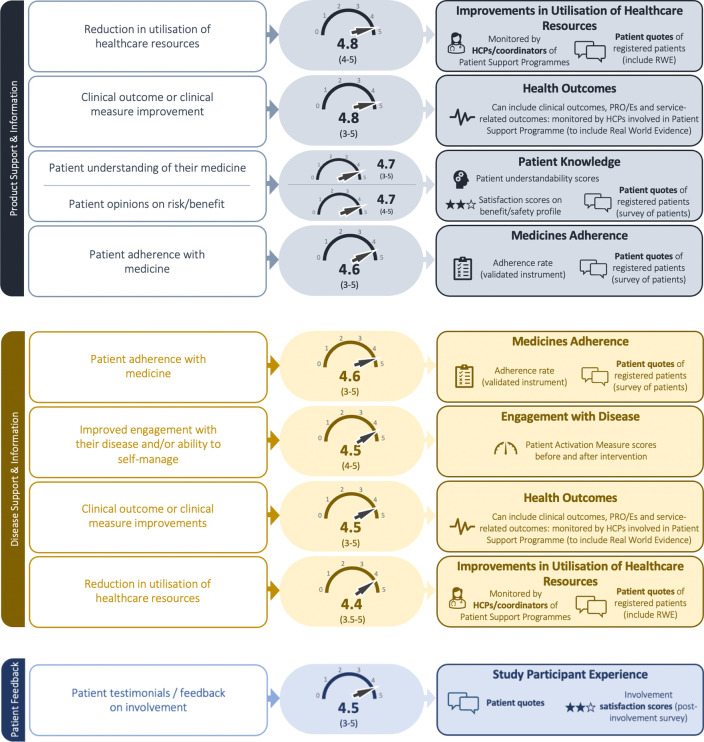
Revision of impact measures

After the workshop, researchers updated the impact measures in light of the agreements made with Working Group patient advisors. A new report was prepared displaying the updated impact measures each with a short summary of the discussions. This report was sent back to the patient advisors to check for accuracy of interpretations and to remain faithful to workshop discussions. Over a series of offline communications (email correspondence) with patient advisors, a consensus was achieved on final wording for each measure (few small changes were made). Figure 2 now displays the final set of impact measures along with corresponding initial versions along with interviewees average importance scores and ranking.

## Results

The geographical, therapeutic area and characteristics of PO and Patient Expert participants can be seen in Table 5.

### Patients’ perceptions and experiences of PEPs

#### Desire to bring about change

Participants commonly expressed the view that aspects of patient involvement in the medicine development process were either inadequate or deficient. This perspective is a strong personal motivator to bring about necessary change within the industry and included frustrations with industry’s patient engagement practices (e.g. burdensome contracts; engaging with the patient community ‘late’ in the medicine life cycle; long-term sustained relationships seldom experienced). There were also particularly notable calls to capture the day-to-day impact of a disease and to utilise patient reported outcome (PRO), patient reported experience (PRE) and quality of life measures in clinical trials (Table 6):

Quality of life is my passion…when you’ve got a treatment then other things start to go, ‘what has this done to my sexual life, to my urinary incontinence’…those metrics are never measured as a primary endpoint… [Participant 6]

**Table 6:** Interview participants mapped against the core recruitment criteria

	Leadership	Representative	Capabilities	Impact	Collaboration
Participant 1	•	•	•		•
Participant 2	•	•	•	•	•
Participant 3		•	•		•
Participant 4	•	•	•	•	•
Participant 5	•	•	•	•	•
Participant 6	•	•	•	•	•
Participant 7	•	•	•	•	•
Participant 8	•	•	•	•	•
Participant 9		•	•	•	•
Participant 10		•	•		•
Participant 11	•	•	•	•	•
Participant 12	•	•	•		•
Participant 13	•	•	•	•	•

Participants did speak positively about ‘mutual benefits’ being established from the start and welcome companies making contact *“proactively…trying to broaden their engagement”* [Participant 4] with POs. Although there was limited discussion about experiences of companies dictating project agendas, there is a sense that POs are now more keen to ensure that all collaboration is aligned with their own strategy, vision and mission*.*

#### Ensure patient voice is heard

Participants’ attitudes and viewpoints are also clearly rooted in a deep empathic understanding of patients’ perspectives with many drawing on their own experiences. They are keen to represent their patient community’s views and where there is routine under-representation of patients this sentiment held greater significance e.g. neuroendocrine cancer or in regions of the world where the patient voice may be less well developed e.g. Malaysia and Latin America:

Where I work, it is not very democratic to have these insights from patients…in places like Latin America, patient voice is not recognisable, not valued. [Participant 12]

#### Feeling accomplishment and ownership

Many interview participants displayed a deep sense of accomplishment where involvement has led to significant milestones being achieved e.g. *“success of Phase 2, where we managed to find the dose of the drug”* [Participant 5]. Entwined with ‘accomplishment’, is a sense of ‘ownership’ characterised by POs being able to bring innovative ideas to a pharmaceutical company to collaborate on such as disease awareness and education campaigns for rural-based physicians.

#### Perception of Industry’s motives to engage with patients

PO representatives had some negative perceptions of companies’ underlying motives to engage with them and are quick to point out what they perceive to be less meaningful *“tick-box”* [Participant 6] involvement e.g. asking a PO to proofread a lay summary without engaging in dialogue or offering any subsequent feedback. Similar scepticism and disappointment are noted after companies withdraw support shortly after a medicine loses patent.

### Perceptions of impact measurement

#### Impact conceptualised as useful outcomes

During discussions about ‘impact’ from involvement activity, participants invariably pointed to a variety of favourable and tangible outcomes that appears to be conceptualised both quantitatively and qualitatively. One participant in Argentina, for example, spoke about an increase in the number of patients correctly receiving a diagnosis following a physician awareness campaign. Another US-based participant cited the number of changes made to a study protocol and reducing the number of secondary endpoints from 10 to two. More qualitative conceptualisations comprise descriptive ‘interpretations’:

…we had 6 meetings, and we reached 100 people or whatever, but that’s very much a series of numbers, whereas impact is probably an interpretation of those numbers. So, we had 6 meetings but actually out of that x, y, z happened, and it was really positive. [Participant 4]

Participants wish to influence change ultimately delivering real and tangible outcomes following any involvement activity, and they are keen to know *“what change they have actually created”* [Participant 8]. A sense of urgency, and empathy is displayed as participants discussed: the impact of Community Advisory Boards they had helped set up; contributions made to lay language disease information; coordinating delivery of educational events for patients. One participant summarised this sentiment particularly well:

…how would this affect patient outcomes, not just it’s a nice-to-have, actually what’s the tangible? what is this going to actually achieve? and I don’t want to achieve it in 20 years, I want to achieve it now. [Participant 7]

Impact on involved patients themselves was also discussed. Some see lessons they have learned and the opportunity to network with other PO representatives as useful ‘personal’ impact. A French participant described learning from a *“more experienced advocate”* [Participant 3] when asked by a company to write a lay language summary for a clinical trial. Related themes of accomplishment, a sense of usefulness and satisfaction were also noted during these discussions.

Additional, unprompted references to impact of involvement activity also included *“how [Industry’s] attitudes have shifted”* or Industry *“learn[ing] about how to use the language of the patient community”* [Participant 8]. Many participants are keen for companies to *“know the emotional story”* [Participant 2] in order to better engage with the patient community.

#### Industry not taking the lead on early impact discussions

This study shows limited evidence of early discussions or expectations being set by pharmaceutical companies about potential impact from PEPs. Participants recognised this as an important first-step discussion that needs to happen:

‘…we pledge that internally we’re going to use the feedback to change the approach to this, or consider our approach to that, or to educate our colleagues about whatever it might be’

Generally, what you would get is just the contract outlining your role and your responsibilities. [Participant 4]

Although there are a few examples of more advanced and structured collaborations, most participants did not readily invite unprompted accounts of companies establishing an early ‘impact agenda’. Instead, invariably PO representatives provided their own notions why patients’ perspectives need to be captured. A US participant outlined a number of innovative ways in which her PO engages with pharmaceutical companies and emphasised the need for patient insights to impact decision-making:

When you talk about patient engagement, it’s one thing to be able to provide the patient voice but it’s really important for us to understand how the regulatory people weigh that…we want to know that it’s useful, otherwise it’s an exercise in futility. [Participant 11]

#### Limited impact measurement and feedback

Most participants state that they receive no formal feedback and follow-up communication of how their involvement in PEPs have led to changes or the insights generated have been incorporated into company decision making. Frustrations are felt particularly strongly where new insights had clearly been generated yet patient contributions are never to be utilised. One participant notably uses the metaphor of a ‘black box’ to describe Industry’s absence of follow up dialogue:

There was one project, there was a round of Patient Experts…it was great work and I was convinced in the beginning that this project would have a high impact…‘never’ heard anything…they do it in a black box and they store it a big warehouse.. [Participant 9]

If you’re asking me to rate from all the impact you have given over the years – may be 30% has had an impact and the rest I have a feeling has gone, it’s somewhere up in the air. [Participant 6]

Some companies only provide feedback reactively i.e. when POs request follow up information. Impact measurement, however, seems more likely to occur where there are larger-scale, longer-term and deeply established relationships developed e.g. the UK-based participant had a particularly strong relationship with one pharmaceutical company that was oriented around a single medicine and rare disease with a dedicated team attached to the PO.

### Commentary on feedback and revisions to impact measures

This section presents an overview of interview participants’ feedback and subsequent discussions with workshop advisors that led to a series of revisions to the original 23 impact measures. Figure 2 displays the original and revised impact measures, and their corresponding importance scores.

#### Medicines R&D priorities

For this first phase of the medicine development process, four impact measures were primarily discussed during interviews. *Development of Patient Reported Outcomes and Experiences* and *Development of tolerability/side effects profile* received the highest average importance scores (4.9 and 4.6, respectively) each with a relatively small range (4–5). Only supportive comments were made during interviews, for example, patient reported outcome and experience measures are widely considered *“most important”* [Participant 10] with one participant vigorously defending them while providing a critique of clinical endpoints: *“if the patient doesn’t feel well, it doesn’t matter what those numbers say, who knows that those numbers are even the correct numbers”* [Participant 13]. Similarly, for tolerability/side effects profile, one participant deemed this *“critically important, patients and families want to know ‘what am I getting into?’”* [Participant 11]. Workshop advisors also held these two measures in high regard. A detailed account of ‘how’ patients influenced the development of patient reported outcome and experience measures and the development of a tolerability/side effect profile was recommended.

Participants were most conflicted with *Number of changes made to the research or development plan* (widest range of importance scores: 2–5 with an average score of 4.4). Supportive comments from interview participants included reminders that poorly designed research results in *“wastage”* [Participant 7] and that therefore involving patients in clinical trial design is *“critically important”* [Participant 13]. Those selecting a low importance score highlighted a potential bias in the measurement of a crude ‘number’ of changes to the research or development plan. They reasoned that the number of changes required is ultimately linked to the quality of the plan ‘before’ patients first have a chance to review it. Therefore the number alone is potentially meaningless unless there is an accompanying description of the changes made. This was echoed during Working Group discussions. Therefore, additional qualitative context has been built into the revised impact measure in the form of descriptions of the type of changes made along with post-involvement patient quotes.

*Development of clinical outcome/clinical measures* scored an average of 4.3 on the importance scale. A number of interview participants suggested patients may be *“restricted”* [Participant 4] and unlikely to *“influence”* [Participant 3] development of clinical study endpoints. However, workshop advisors reinforced that both the measure and patients recognised as key stakeholders here are indeed critical since *“choosing the wrong primary endpoint can ruin a trial”* [Participant 13]. Greater qualitative context and gaining an understanding of patient influence was incorporated into the revised version.

#### Clinical trial design

For the next stage of the development process, clinical trial design, *Study participants’ experience and satisfaction ratings*, received the highest average importance score (4.7) and smallest range across interview participants (4–5). Largely supportive comments were made during interviews and the workshop, *“Absolutely, I think that informs what we do next”* [Participant 13].

One participant, with extensive experience setting up and co-managing clinical trials with a pharmaceutical company, highlighted due consideration be given to potential *“confounding variables”* [Participant 8]. Poor hospital experiences, for example, could affect study participants’ overall satisfaction scores which may support incorrect conclusions. For this reason, specific study participant experiences in the form of qualitative quotes have been included in the revised version.

Although *Number of changes to reduce the burden of study for patient participants* was considered of high importance (average score: 4.5), a few participants provided an importance score of 3. Once again, capturing only the ‘number’ of changes was considered insufficient. Instead, *“it’s about the quality of changes…the ability to change…it’s a qualitative measure”* [Participant 13] therefore a description of changes and patients’ quotes were incorporated into this impact measure.

*Number of patients complying with study protocol* received two particularly low importance scores (2 and 2.5) from participants involved in therapy areas in which study participants typically do not dropout in (bladder cancer and a rare genetic disease). Since R&D metrics already capture data on non-compliance, workshop discussions recommended a shift in the focus for this impact measure from compliance to non-compliance or withdrawal from the study. Additional quantification was advised, namely, both ‘number’ and ‘percentage’ of study participants not complying. It was also felt that subjective feedback from study participants was needed in order to learn for future research programmes.

The lowest average importance score (3.6) was received for *Number of changes made to final version of patient-facing documents* and the widest range (0–5). The one individual scoring this as a 0, along with a number of others once again took issue with the lack of insight achieved and relevance of only capturing the ‘number’ of changes: “*I don’t think the numbers matter, it matters that this really educates…”* [Participant 11]. Greater descriptive context and capture of the degree to which patients influenced the development of patient-facing documentation was incorporated into the final version.

From the five impact measures, one impact measure was removed: *Earlier regulatory submission, approval and/or market access submission.* Interview participants rated this relatively lower and questioned the inclusion within the Clinical Trial Design category of measures. One interview participant expressed uncertainly about the link between patient involvement and early submissions and approval. Others felt this measure was of more immediate relevance to companies or that the influence patients are likely to have is limited.

It’s important for the company, not important for the patient…patients want to have a drug that’s working and has come through all the safety rules… [Participant 6]

Workshop advisors unanimously agreed to remove this measure from the Clinical Trial Design category seeing it more as an internal company key performance indicator (KPI).

#### Regulatory and market access submissions

*Patients’ critique of evidence generated from clinical trials included in submission* received an average importance score of 4.5. Participants and workshop advisors were strongly in favour of retaining this impact measure and felt, in this case, a binary outcome (i.e. yes = included and no = not included) was sufficient. Advisors recommended a change to terminology from ‘critique’ which was considered too subjective to ‘evaluation’. Similarly, *Patient insights included in development program to inform submissions* also remained a binary outcome measure. With this impact measure, mixed opinions were noted. While some acknowledge *“regulatory bodies want to see that, especially in rare diseases where you’re not getting huge randomised controlled trial numbers”* [Participant 7], others caution that *“scientists deal with the numbers…patients don’t really make much difference”* [Participant 1]. However, workshop advisors unanimously agreed that the impact measure should be retained, that patients are in fact instrumental and that such involvement was already a requirement in many countries.

There was widespread support to measure the extent to which patients involved at this stage help to ensure that regulatory and market access review and approval are *consistent with the patient population studied*:

Yes, especially in our population [neuroendocrine cancer] because they all respond differently, some get a great response, others don’t, that doesn’t mean you just ignore those ones [Participant 7]

For this measure, gaining a wider understanding in the form of patients’ quotes and satisfaction scores was proposed.

For the final measure, workshop advisors felt there was potential ambiguity where ‘*more informed label’* was used asking the question ‘relative to what?’. Instead, there was greater preference for the terms ‘patient-friendly’ and ‘benefit/safety profile’. Once again, satisfaction scores were felt more useful rather than merely capturing whether patient involvement occurred or not.

#### Product support and information

The next two categories of impact measures fall under Product Support and Information and Disease Support and Information. In both categories, *Clinical outcome or Clinical measure improvements* and *Reduction in utilisation of healthcare resources*, repeatedly scored high average importance scores (between 4.4 to 4.8). On the former, and echoing an earlier point, a participant emphasised the importance of patient reported outcome/experience measures: *“…sometimes clinical outcomes is not as important as the overall functionality, being able to work, that kind of thing”* [Participant 4]. This triggered a wider discussion during the workshop about what constitutes *Health Outcomes.* It was subsequently agreed that this measure should incorporate impact of patient involvement on three additional aspects that collectively fall under Health Outcomes i.e. clinical outcomes; patient reported outcomes/experiences; and service/infrastructure-related outcomes. Discussing *Reduction in utilisation of healthcare resources*, one participant stated:

I think this is fabulous…I think we don’t think enough about patient engagement with payers and what it ultimately means to the healthcare system at large… [Participant 8]

In addition to a numerical appreciation of the utilisation of healthcare resources, research feedback also supported the inclusion of qualitative patient quotes.

A third impact measure that also appears in Disease Support and Information is *Patient Adherence with the medicine.* During the interviews this consistently received high importance scores (average: 4.6) however one participant’s views on the concept of ‘adherence’ generated significant discussion and revision of the measure during the workshop with the emphasis shifting from the ‘patient’ to the ‘medicine’:

I struggle with this; I don’t like the word ‘adherence’. There’s so many things tied to it. Adherence is a loaded term…because there’s systemic issues… [Participant 8]

One workshop patient advisor argued:

Patients generally want to be adherent. The way this statement reads puts the blame on the patients. Medicines fail patients, not the other way around. A good medicine, good clinical support, good education about the medicine, promotes adherence.

There was a general consensus during the workshop that unless adherence was measured using a robust, validated instrument, there is a risk that adherence measurement could be a highly subjective process. Conversely, it was also important to capture individual reasons (qualitatively) for non-adherence so that they can be addressed.

*Patient opinions on risk/benefit* and *Patient understanding of their medicine* both achieved average importance scores of 4.7. Workshop advisors proposed combining the two into: *Patient Knowledge*. Advisors also noted it was important that information supplied to patients registered on patient support programmes was in lay language, comprehensible and patient-friendly. For this reason, the *Patient Knowledge* impact measure incorporates capture of patients’ understandability scores, satisfaction scores and patients’ quotes.

#### Disease support and information

There was wide support and high importance scores given to the impact measure: *Improved engagement with their disease and/or ability to self-manage.*

I would put that really high…you need to realise that it actually had some kind of positive effect. [Participant 9]

Workshop advisors echoed this sentiment and agreed on the use of the Patient Activation Measure (PAM) to assess patients’ knowledge and level of confidence with managing their condition before and after the patient support programme [46].

#### Patient involvement feedback

A final impact measure, *Patient testimonials/feedback on involvement*, could be applied to each stage of the medicine development process. Many participants considered this a crucial measure:

Really important. I think unless you’re collecting that, what’s the point! [Participant 4]

Workshop discussions addressed a number of issues. Capturing such feedback was seen as a way to learn and improve future PEPs: *“patients listen to other patients”*. Another advisor cautioned against acquiring only a superficial understanding and that attention ought to be given to robustly capturing such feedback otherwise there is a risk that insights are *“misinterpreted and misused”*. Finally, advisors agreed to remove the word ‘testimonial’ as the focus with this impact measure is to record both positive as well as potentially negative experiences of involvement. The revised measure, *Patient Feedback*, now incorporates satisfaction scores and patient quotes captured post-involvement.

## Discussion

### Reflections on findings

Patient advocates collaborating with biopharmaceutical companies are driven by a unique set of insights, expertise and experience of living with a medical condition to deliver meaningful patient-centric impact. PO representatives involved in PEPs want the patient’s voice to be heard, particularly where there is historic under-representation. They are very much independent stakeholders committed to delivering on their own organisations’ vision and mission. Therefore, PEPs need to be aligned with POs’ goals and profiles of the new category of experts–patient experts. Establishing mutual benefits are typically encouraged and indeed celebrated.

These perspectives and the PO’s mindset underpin their considerations on impact and impact measurement. Indeed the revisions made to the impact measures in Table 2 clearly drew on such experiences and the empathic understanding that they engender. It’s important to note that the revised impact measures aligned to and reflect the good practices that are starting to emerge across R&D: patient reported outcomes and experience as endpoints in clinical trials, more holistic consideration of health outcomes and patient-centred outcomes (PCORI), broad implementation of patient support programmes (post-authorization) and services (within the clinical operations), advice-seeking and insights gathering activities, non-interventional studies and registries, initiatives to enhance scientific transparency across R&D [8, 13, 15, 17, 18, 23, 26, 31, 36, 38, 48, 49]. The study has shown that measuring impact is clearly important to patients and POs because knowledge of achieving tangible patient-centric outcome instils a sense of accomplishment, ownership and accountability.

The study sheds light on how patients are likely to conceptualise ‘impact’. Patients typically describe impact as a positive, tangible or useful outcome—in other words—delivering ‘value’ important for them. The authors of this paper therefore propose the term ‘value-impact’ to comprehensively characterise this conceptualisation. There is also an appreciation for the subjective and multi-faceted nature of ‘impact’. For instance, one can expect to see direct impact on a PEP’s underlying objectives; however, there is also impact on the involved patients themselves or ‘personal gains’ (education and networking) from taking part in these projects and a ‘maturing’ impact on Industry e.g. cultivating a shift in culture and attitudes, understanding the emotional story and developing an appreciation for the language and voice of a patient community.

The revisions made to the impact measures fall into three categories: (1) greater context required (2) ensure nature of patient influence is captured. (3) terminology changes. By far, the greatest number of revisions fall into ‘requiring greater context’. For some measures, this meant additional quantitative capture e.g. capturing ‘percentage’ as well as ‘number’ of study participants not complying or withdrawing from a study. For the most part, however, patients called for additional qualitative context, such as descriptions, patient quotes and satisfaction. The parallels between the patient-centred method and qualitative inquiry have been acknowledged previously [47]; both adopt a holistic, naturalistic and empathic understanding of the phenomena being investigated. This tendency for a deeper and more nuanced understanding is also seen in patients’ strong preference to be involved in the development of PROMs and Patient Reported Experience Measures (PREM), recording the ‘day-to-day’ experience of their condition and the value delivered by a medicine. The support for the ‘Patient Feedback’ impact measure is a further endorsement of a more descriptive understanding of the impact of involvement in PEPs. It should be noted, though, that patient participants certainly did not state that quantitative data capture and insights were not required or considered less valuable; instead, there was a sense that they are not only interested in the ‘how many’ but also in the subjective and interpretive reporting of impact e.g. capturing patients’ experiences of newly developed patient support programmes or appropriately contextualising a poor hospital visit experience during clinical study participation that may otherwise unfairly reflect on the biopharmaceutical company’s broader research program. There was also a sense that there is greater learning to be derived from qualitative capture both to improve future PEPs and for the underlying project objective. This call for ‘greater context’ reinforces Vat et al.’s [23] recent work who concluded that, in fact, a “set of coherent measures” is needed rather than the application of single measures. The present study now provides the rationale for this claim, crucially, from the patient’s perspective.

Secondly, it was considered important for some measures to be able to define how patients ‘influenced’ key aspects of early R&D and clinical trial design. While this is arguably another example of qualitative insight, the authors considered this revision to be worthy of separate and distinct acknowledgement. Here, internal R&D personnel would provide an objective description of how patients influenced Target Value Profile (TVP) development, asset development plan/strategy and clinical trial delivery e.g. in the development of patient-facing documents or development of clinical outcomes. In addition, patient post-involvement feedback will capture patients’ opinions and sentiment about the degree to which they felt they were ‘genuinely’ able to influence decision-making processes. This corresponds well with earlier themes of accomplishment and ownership arising from co-creating tangible impact.

Finally, a few of the impact measures originally contained words or phrases that patients felt were less patient-friendly and recommended alternatives e.g. Patients’ ‘critique’ of evidence… amended to Patients’ ‘evaluation’ of evidence… and ‘Patient adherence’ to ‘Medicine adherence’. This last change demonstrates how patient insight into co-developing impact measures can reorientate biopharmaceutical companies’ focus, here shifting the emphasis to developing medicines and support programmes that patients value and that enable adherence rather than attributing lack of adherence solely to patients’ behaviour.

The PARADIGM group’s recently published framework outlines a comprehensive set of measures, complete with suggested methods of measurement that biopharmaceutical companies should now consider as part of their patient engagement monitoring and evaluation plans [24]. Although the present study does not identify new measures, it does illustrate how PO representatives consider and prioritise impact measures at various stages of the medicines life cycle. In addition, many of PARADIGM’s 87 metrics (of which 45 are categorised as ‘impact metrics’) do resonate well with the revised impact measures outlined in Table 2. For instance, the themes discussed above are also apparent in the PARADIGM framework: greater qualitative context e.g. for clinical study participants’ experience (accessed through reflection sessions), capturing how patients were able to influence decisions, and collecting data on personal/professional skills (i.e. personal gains).

### Implications for future practice and research

We have heard that patients expect a meaningful relationship with the biopharmaceutical industry, along with transparent dialogue before, during and after a PEP. Discussions about planned impact must take place much earlier, ideally at project initiation, with expectations set for all stakeholders. The *“black box”* [Participant xx] metaphor one interview participant used was particularly notable and reflects a general sentiment heard from other participants on the lack of project feedback, which ought to include the extent to which value-impact was delivered, incorporated into decision-making and operationalised. Biopharmaceutical companies therefore need to ensure there is ‘proactive’ feedback of achieved impact, not reactive, to avoid partnered POs feeling frustrated about potential wasted opportunities and to avoid damaging the establishment of sustainable, productive, good working relations. Therefore, establishing long-term, sustainable partnerships with patient experts and POs is another critical success factor.

This study provides additional considerations for the development of new impact measures: they should be tailored to the stage of a medicine’s life cycle in which PEPs are being undertaken; should be co-developed with patients with efforts to ensure appropriate context is captured, how patients influenced impact and the right, patient-friendly terminology is used. Continuous feedback from involved patients is also critical as it provides opportunities to learn and improve for future PEPs. Having co-developed a list of impact measures, the next step for biopharmaceutical companies is to consider how best to implement their utilisation. This practical step should include cross-functional and functional considerations on how the suggested impact indicators could be embedded to the existing systems and operational excellence dashboards, internal piloting and validation, communication and training to be delivered to personnel. The other logistical considerations may be developing new qualitative and quantitative data collection and tracking tools, assigning new roles and responsibilities within the company and developing data visualisation both internally and externally.

It will be important to further involve patients in the implementation process ensuring, for instance, newly (co-)developed data collection and tracking tools to capture the various aspects of ‘value-impact’. The researchers are keen to work collaboratively with other industry representatives and stakeholders sharing this practice and elaborating the unified standard of PEP impact measurement.

### Strengths and limitations

Our methodology has consisted of systematically gathering the perspectives of patients during initial considerations of impact measures as well as in subsequent reflections and interpretations. The conclusions drawn from this study are therefore not only those of the researchers but represent the views of the patient advocate co-authors who were deeply involved throughout all stages of our research from planning to publication. This level of co-creation with patients on a publication on patient-centric impact, to our knowledge, does not exist. We also employed a two-phased research methodology (semi-structured interviews and group discussion during the workshop), which supports the claim that the revisions made to the original set of impact measures have been carefully and thoroughly considered.

An additional strength to our study is the consideration of impact measures across a number of stages of the medicine’s life cycle. Few authors have looked beyond patient involvement in Clinical Trial Design, to incorporate Medicines R&D Priorities and Regulatory and Market Access Submissions [23, 38]. We have also considered PEPs and corresponding patient-centric impact measures in a post-authorisation setting, namely Patient Support Programmes (Product and Disease Support & Information). The patient engagement frameworks and conceptual models we considered as prototypes of the proposed impact measures were developed for broad application and significantly informed authors during the initial review of progress to date in the area.

Limitations of this study must also be acknowledged. A relatively small and non-random, purposive sample of interview participants and workshop advisors was used. This is common practice within the qualitative research paradigm, however we do recognise the potential for selection bias and cannot make claims to generalisability. While the authors hope that the rich descriptions and illustrative quotes provide readers with enough context to consider the ‘transferability’ of findings to other settings, there is certainly scope to survey a larger population of PO representatives in order to build the evidence base for broader application.

Another limitation with this study that is also a characteristic of qualitative research is the ‘interviewer effect’ and the inherent bias arising from the interviewer’s own experiences and background. Both interviewers (researchers on this study) have a history of working with POs and generating patient insights that could have influenced the way questions were asked and indeed, the subsequent interpretations and identification of themes. Reflexivity, the practice of being aware of one’s own experiences and positionality in order to remain ‘intentionally’ objective during the interviews, was discussed by the interviewers prior to meeting with participants. Indeed, one of the reasons to have two interviewers/researchers was to ensure there was more than one coder and thereby help improve the rigour of qualitative analysis.

It should also be noted that the research process started with a pre-defined list of impact measures rather than a blank canvas. Although this was clearly done for pragmatic reasons and in recognition of previous efforts and earlier debates around several conceptual frameworks and models, there is a risk that interview participants may have been biased by being introduced to an ‘existing set’ of measures. Therefore, efforts were made to clarify that these impact measures are only initial prototypes and were actively encouraged to critique/correct their individual utility as well as suggest new measures.

The proposed set is not exhaustive and should also be considered as a generic list of measures that, alone, may not always be able to address real world healthcare challenges, specific requirements and unmet needs of cohort/group of patients living with a particular medical condition(s) and their involvement in specific PEPs. Therefore, the authors accept that, for a given situation, it may be that only a limited number of the measures in Fig. 2 can be applied and will require supplementing with other, more relevant measures.

Finally, the study methodology incorporated a small quantitative element into the delivery of the interviews. Impact measures were scored on a 0–5 importance score. Due to limited sample size, the subsequent average importance scores are not generalisable (for reasons discussed above) as may be expected in standard quantitative research where larger, representative samples would be used. The averages were therefore taken only as ‘indicators’ to facilitate discussions during the workshop.

## Conclusion

This qualitative study explored five stages of the medicines life cycle and provides insights on what impact measures are considered important to PO representatives and why. While operating against a backdrop of agreed mutual benefits, they are deeply motivated by their POs’ own set of vision, mission and goals as well as their personal experiences that underpin their perceptions and expectations of delivering ‘value-impact’ from PEPs. The study reveals a deep sense of ownership and immersion that PO representatives feel when collaborating on PEPs. For this reason, the vast majority of impact measures were revised to incorporate wider context through greater data capture, primarily in the form of relevant contextual descriptions, patient quotes and the degree to which involved patients truly influenced decision making.

The authors hope by understanding the subtleties of how PO representatives conceptualise impact, outlined in this paper, and in light of other recently published work, there is a real chance to see wide-scale adoption and implementation of measurement of value-impact across the biopharmaceutical industry. Further research is warranted on a value-impact measurement plan, which the authors of the current study also aim to conduct and publish in due course.

## Data Availability

The datasets generated and analysed in the current study aren’t publicly available due to participant confidentiality, but are available from the corresponding author upon reasonable requests from qualified researchers who provide a valid research question.

## Abbreviations

ECPC: European Cancer Patient Coalition
EMA: European Medicines Agency
EORTC: European Organisation for Research and Treatment of Cancer
EUPATI: European Patients’ Academy on Therapeutic Innovation
FDA: (US) Food and Drug Administration
HRQoL: Health-related quality-of-life
HTA: Health Technology Assessment
IKCC: International Kidney Cancer Coalition
INCA: International Neuroendocrine Cancer Alliance
MHRA: (UK) Medicines and Healthcare products Regulatory Agency
NHC: (US) National Health Council
PAM: Patient Activation Measures
PEP: Patient Engagement projects
PFMD: Patient Focused Medicine Development
PO: Patient organisation
PRE: Patient reported experience
PREM: Patient Reported Experience Measures
PRO: Patient reported outcomes
PROM: Patient Reported Outcomes Measurements
R&D: Research and development
RCT: Randomised controlled trial
TVP: Target Value Profile
UK: United Kingdom
US: United States
VBA: Value-based assessment
WAPO: World Alliance of Pituitary Organisations
